# Communication between parents diagnosed with cancer and their children: study with data triangulation

**DOI:** 10.1590/1980-220X-REEUSP-2023-0079en

**Published:** 2024-02-19

**Authors:** Estela Ferreira da Silva, Maria das Graças Silva Matsubara, Mariângela Abate De Lara Soares, Maria Cristina Mazzaia, Edvane Birelo Lopes De Domenico

**Affiliations:** 1 A.C. Camargo Cancer Center, São Paulo, SP, Brazil.; 2 Universidade São Judas, Santos, SP, Brazil.; 3 Universidade Federal de São Paulo, São Paulo, SP, Brazil.

**Keywords:** Communication, Parent-Child Relations, Medical Oncology, Expressed Emotion, Health Education, Comunicación, Relaciones Padres-Hijos, Oncología Médica, Emoción Expresada, Educación en Salud, Comunicação, Relações Pais-Filho, Oncologia, Emoções Manifestas, Educação em Saúde

## Abstract

**Objective:**

To characterize the perceptions and feelings of parents diagnosed with cancer in relation to communication with their children between 3 and 12 years old.

**Method:**

A cross-sectional, multicenter, with data triangulation, through structured and semi-structured interviews, with a question with a Semantic Differential Scale, carried out with the father or mother with cancer undergoing outpatient treatment in two hospital institutions in the city of São Paulo, São Paulo, Brazil. Data were analyzed using descriptive statistics, content analysis, using the ATLAS.ti 8.0R software and the Social Representation Theory.

**Results:**

Forty-three respondents participated, 37 (86.0%) were female, 23 (53.5%) aged between 31 and 50 years old, 29 (67.5%) with only children between 7 and 12 years old. The experience was considered painful (73.1%), stressful (53.6%), clear (53.7%) and safe (51.2%). The feelings experienced generated two categories: Trial by fire; and Grateful rewards. Children’s reactions from parents’ perspective generated the categories: Sadness and suffering; Trust and support; Change of behavior; and Denial or insensitivity.

**Conclusion:**

Communication was assessed as negative and conflicting, positive and welcoming, and causing changes in children’s behaviors.

## RESUMO

**Objetivo:**

Caracterizar as percepções e os sentimentos dos pais diagnosticados pelo câncer em relação à comunicação com seus filhos entre 3 e 12 anos.

**Método:**

Transversal, multicêntrico, com triangulação de dados, por meio de entrevistas estruturadas e semiestruturadas, com uma pergunta com Escala de Diferencial Semântico, realizadas com o pai ou a mãe com câncer em tratamento ambulatorial em duas instituições hospitalares da cidade de São Paulo, São Paulo, Brasil. Os dados foram analisados por meio da estatística descritiva, análise de conteúdo, utilizando o *software* ATLAS.ti 8.0^R^ e a Teoria das Representações Sociais.

**Resultados:**

Participaram 43 respondentes, sendo 37 (86,0%) do sexo feminino, 23 (53,5%) com idades entre 31 e 50 anos, 29 (67,5%) com filho único entre 7 e 12 anos. A experiência foi considerada dolorosa (73,1%), estressante (53,6%), clara (53,7%) e segura (51,2%). Os sentimentos vivenciados geraram duas categorias: Prova de fogo; e Grata recompensa. As reações dos filhos, na perspectiva dos pais, geraram as categorias: Tristeza e sofrimento; Confiança e apoio; Mudança de comportamento; e Negação ou insensibilidade.

**Conclusão:**

A comunicação foi avaliada como negativa e conflituosa, positiva e acolhedora, e causadora de mudanças nos comportamentos dos filhos.

## RESUMEN

**Objetivo:**

Caracterizar las percepciones y sentimientos de padres diagnosticados con cáncer en relación a la comunicación con sus hijos entre 3 y 12 años.

**Método:**

Transversal, multicéntrico, con triangulación de datos, mediante entrevistas estructuradas y semiestructuradas, con una pregunta con Escala Diferencial Semántica, realizadas con el padre o la madre con cáncer en tratamiento ambulatorio en dos instituciones hospitalarias de la ciudad de São Paulo, São Paulo, Brasil. Los datos fueron analizados mediante estadística descriptiva, análisis de contenido, utilizando el software ATLAS.ti 8.0^R^ y la Teoría de las Representaciones Sociales.

**Resultados:**

Participaron 43 encuestados, 37 (86,0%) eran del sexo femenino, 23 (53,5%) con edades entre 31 y 50 años, 29 (67,5%) con hijos únicos entre 7 y 12 años. La experiencia fue considerada dolorosa (73,1%), estresante (53,6%), clara (53,7%) y segura (51,2%). Los sentimientos vividos generaron dos categorías: Prueba de fuego; y recompensa agradecida. Las reacciones de los niños, desde la perspectiva de los padres, generaron las categorías: Tristeza y sufrimiento; Confianza y apoyo; Cambio de comportamiento; y Negación o insensibilidad.

**Conclusión:**

La comunicación fue evaluada como negativa y conflictiva, positiva y acogedora, y provoca cambios en las conductas de los niños.

## INTRODUCTION

Cancer is a chronic disease with global incidence that affects people of all age groups, of both sexes. Unfortunately, it is still socially stigmatized, as there is a belief in the imminent risk of death when diagnosed with cancer, fear of aggressive and sometimes mutilating, long-term treatments, exacerbated by fear of pain, feelings of guilt and even the risk of social exclusion^(1)^.

Despite the strong relationship between cancer diagnosis and population aging, there is a considerable incidence of various cancer diagnoses in adults under 60 years of age. In 2012, the World Health Organization (WHO) estimated the occurrence of 348,457 new cases of cancer globally, with 25% of this total in the population between 15 and 44 years old resulting from family histories and also factors such as lifestyle, environmental exposures and generational differences in eating habits^(1,2)^.

Certainly, cancer diagnosis has different clinical and psychosocial meanings according to the age group affected. In young adults, cancer causes a multitude of negative impacts due to disease diagnosis at the height of their professional and relational potential, with the possibility of having dependent young children, among other aspects of adult life that are still in prospect. Parental cancer diagnosis, i.e., a father or mother with a history of cancer, predicts temporary or permanent changes in the life of a person and their family, restricting or even suspending their activities and projects^(3,4)^.

In recent studies, with representative population samples from the United States of America (USA), families with and without parental cancer were compared, and the results showed greater difficulties experienced by children between 5 and 17 years of age in families with one parent sickened by cancer. Among the difficulties experienced were food insecurity, risk or loss of housing and barriers to transportation and obtaining medical care, especially in low-income families. There were also school absenteeism, increased frequency of need for health care and mental health problems in children with parental cancer in general^(5,6)^.

Once it is characterized that cancer diagnosis affects the functioning of all family members, effective communication between parents diagnosed with cancer and their children is a challenge, and the cultural, sociopolitical, economic and educational context is an important intervening factor, as pointed out by international studies^(7)^.

In the case of parental cancer with children still dependent, the problem takes on a certain amplification that may begin with parents’ difficulty in coping during the diagnostic and treatment initiation phases, aggravated or not by the initial or advanced (metastatic) state of illness. In the diagnostic phase or any other phase of the process of becoming ill with cancer, the tendency to spare children from parental diagnosis is possible and understandable. However, continued dialogue with children can result in problems that express a lack of clarification of the reality experienced, such as increased attachment, aggressiveness/irritability, sadness, withdrawal, difficulties concentrating or difficulties sleeping, among others^(3,7)^.

Given the complexity of the topic, it was found that there is a need for studies in Brazilian society with the aim of understanding the phenomenon in order to formulate guidelines for health professionals and sick parents. Thus, this study aimed to characterize the perceptions and feelings of parents sick with cancer in relation to communication with their children between 3 and 12 years old.

## METHOD

### Study Design

This is a cross-sectional, multicenter study, with data triangulation so that the phenomenon could be validated and understood in a complementary way, based on the selection of different methods, such as qualitative and quantitative, and with the inclusion of respondents from two centers, respecting the characteristics of homogeneity for joint analysis^(8–10)^.

The COnsolidated criteria for REporting Qualitative research (COREQ) checklist steps guided the process of preparing the research project in the qualitative aspect, preponderant in the study^(9)^.

### Study Site

The study was carried out in two large tertiary-level hospital institutions: one with a university hospital profile, the other philanthropic. They are located in the city of São Paulo, São Paulo, Brazil, assisting patients diagnosed with cancer in the Brazilian Health System (SUS – *Sistema Único de Saúde*), a Brazilian system with characteristics of being public and universal.

### Population and Sample

The sample was non-probabilistic, consisting of 43 patients.

### Eligibility Criteria

People over the age of 18 diagnosed with cancer, of any etiology and stage, within a period of less than or equal to two years, undergoing isolated or combined antineoplastic chemotherapy, whether or not they have undergone previous treatments, with biological children and/or foster children aged between 3 and 12, were included. Illiterate people, those with psychiatric disorders or those experiencing auto and allopsychic disorientation, documented in patients’ electronic medical records were excluded.

### Instruments and Data Collection

Data were collected from February 2019 to January 2021, with interspersed periods of interruptions due to institutional guidelines for cessation of in-person research activities, aiming to manage the COVID-19 pandemic, which began in March 2020, which, in practice, generated an increase in the pre-defined time for data collection and also made it difficult to meet again with respondents to validate discursive responses.

The main researcher was the interviewer, trained to collect data by the researcher, with the role of study guide, and who also supervised the process of organizing and processing all data. In data collection, the main researcher was responsible for consulting, the day before the interview dates, the outpatient schedules and creating a list with the names and times of attendance of potential patients. Participants were approached in person upon arriving at the outpatient clinic, checking data that did not appear in the medical record corresponding to the inclusion criteria, and being informed about ethical research procedures.

After accepting and signing the Informed Consent Form (ICF), the interview took place following a protocol that was reproduced equally in both institutions for subsequent data compilation, configuring the multicenter study. The protocol consisted of a personal presentation by the researcher, ICF application, directing respondents to a reserved or private location and application of the three instruments, with a mean duration of 30 to 45 minutes.

Firstly, the structured instrument was applied to sociodemographic and clinical data, containing sex, age, race, education, economic condition, number and age of children, diagnosis, proposed treatment, presence of clinical artifacts and ECOG (Eastern Cooperative Oncology Group Performance Current Status Scale). Data on unanswered items were obtained from patients’ electronic medical records.

Subsequently, the semi-structured instrument was applied, whose questions were prepared based on an integrative review study on the topic^(11)^, namely: did you talk about the cancer diagnosis with your partner? children? Who took responsibility for talking about your illness with child(ren)? At what time? Did you receive help from anyone? What degree of relationship did you have with this person? How do you assess this person’s help? How do you assess the experience of talking to your child? How do you assess your child’s feelings and behaviors based on this conversation?

Finally, the Semantic Differential Scale (SDS) was applied, consisting of adjectives presented from the most negative to the most positive. The adjectives were chosen considering the intention of characterizing parental feelings when communicating disease diagnosis. In its constitution, the choice of adjectives, the order of scales and polarity were kept constant for all concepts, as advised in specialized literature^(12,13)^. The introductory question for choosing adjectives was: what was the feeling experienced when communicating with your child about the diagnosis of your illness? Select the words and intensity that best express the feeling. The adjectives (descriptors) were presented in subscales with opposite meanings, namely: uncomfortable - comfortable; painful - painless; complex - simple; unsafe - safe; confused - clear; stressful - not stressful; tiring - not tiring. For each adjective, participants chose between negative values (−3, −2, −1, decreasingly) and positive values (1, 2, 3, increasing), with zero being the neutral point.

The set of three instruments was applied in a pilot test to assess comprehensibility. The five participating patients met the eligibility criteria. The instruments were offered to be completed by participants, in a private environment, after the research was explained. The respondents consented to participate and there was no recording. It was observed that the respondents had difficulty writing and preferred to speak while writing and, therefore, the researcher verified that important information was said and was not written in the instrument. The time varied from 30 to 40 minutes. They expressed understanding of words and linguistic construction of instruments. The pilot test justified the choice to record all interviews, with the researcher helping to read the questions.

### Data Processing and Analysis

Quantitative, sociodemographic and clinical data were organized and presented with descriptive statistics and tables. The data from EDS were displayed in boxplot graphs to visualize the distribution and discrepant values of the adjectives as well as to facilitate comparison between them^(14)^.

For qualitative data, Bardin’s content analysis technique was used^(15)^. The recorded responses were transcribed into Word^R^ files for each participant identified as P and cardinal numbers. Due to interruptions in the data collection period due to the COVID-19 pandemic, the stage of confirming the transcribed data with the participants themselves became unfeasible, opting for review by two researchers, independently, to ensure fidelity of transcribed contents. Participant files were included in the ATLAS.ti 8.0^R^ (Scientific Software Development, Berlin, Germany, Educational) software, license obtained in 2017.

Together, semantic interpretations and registration units (codes), in which citations (sentences/paragraphs) of participants’ responses were included, were compared and, after consensus, the elements (words) were distributed. The distribution made it possible to count the frequency of elements allocated to the registration units and, from then on, they were represented in a network (networks), which distributed registration units based on the categorical axis.

The interpretation and inferential analysis of participants’ statements were based on the postulates of social representations (SR), which are capable of offering meaning to an action as they unveil the phenomenon, direct the way of interpretation, judgment and decision-making position, giving meaning to behaviors^(16)^.

The choice for this theoretical-conceptual framework emerged from the social meanings of falling ill with cancer which, despite all the accumulated knowledge and innovations of the last three decades, remains a death sentence or a sentence of profound suffering in many contemporary societies, characterizing ambivalences between feelings positive, demarcated mainly by coping, and negative, such as denial and fear^(17)^.

### Ethical Aspects

The study was approved by the Research Ethics Committees of the *Universidade Federal de São Paulo*, under Opinion 2.017.291/2017, and the *Fundação Antônio Prudente*, under Opinion 2.445.168/2017, in accordance with Resolution 466/12, with application of ICF without data collection. Participant confidentiality and anonymity were guaranteed through the code name “Participant”, followed by a cardinal number, which indicates the sequence of the interviews carried out.

## RESULTS

A total of 43 cancer patients undergoing treatment at the antineoplastic chemotherapy outpatient clinics of the participating institutions participated. Regarding respondents’ age, 55.1% were between 21 and 39 years old and 44.1% were between 39 and 59 years old, 86% were female, 55.8% self-declared brown or black, 74.4% married and/or in a stable union and 62.8% had completed high school. In the economic assessment^(15)^, participants were concentrated in economic class C (67.5%), followed by B (25.5%), D and E (7.0%). As for children, 29 (61.7%) had a single child aged between 7 and 12 years and 23 (38.3) had a child aged between 3 and 6 years.

Regarding clinical data, there was a predominance of breast cancer (23; 53.6%), followed by digestive system cancer (6; 13.8%). The majority received antineoplastic chemotherapy and combined therapy (chemotherapy + endocrine therapy or chemotherapy + radiotherapy), underwent surgery (28; 65.1%) and with an ECOG Performance Status Scale between zero and one (38; 88.4%). Additionally, seven (16.3%) were using a fully implanted central venous catheter, in addition to six (13.9%) using a breast expander and one (2.3%) using an intestinal stoma.

Regarding communication about the diagnosis and who was the major player, 41 (95.4%) reported having communicated the illness situation to their children, with 27 (65.7%) communicating in the week or on the day they received the diagnosis. Data can be found in Table 1.

**Table 1:** Temporal moment of communication of diagnosis of parents with cancer with their children about the diagnosis and support received by other people for this communication – São Paulo, SP, Brazil, 2021.

Communication established and support received	N(43)	%
**Who communicated the cancer diagnosis to their child**		
Patient	35	81.3
Another person	6	14.0
Did not communicate	2	4.6
**Time elapsed from diagnosis to communication about the disease**		
On the day	5	12.1
On the week	22	53.6
1 month to 12 months	14	34.1
**Who conducted the conversation**		
Patient	35	81.3
Partner who lives in the same house	1	2.3
Spouse	2	4.6
Spouse and daughter	1	2.3
Sister	1	2.3
Doctor	1	2.3
**Received help with communication**		
Yes	13	30.3
No	28	65.1
**Degree of kinship of the aid received**		
Children	5	2.3
Brother or father	2	9.4
Mother	3	2.3
More than one relative	3	2.3
Patient (did not receive help)	28	65.1
Did not communicate	2	4.6
Total	43	100.0

Only two (4.6%) participants did not communicate their illness to their children. Thus, one man justified the geographical distance, because the children were in another federal state in Brazil, and the other explained the son’s age, claiming that, as he was only 5 years old, he thought the news was “very strong”.

**Figure 1:**
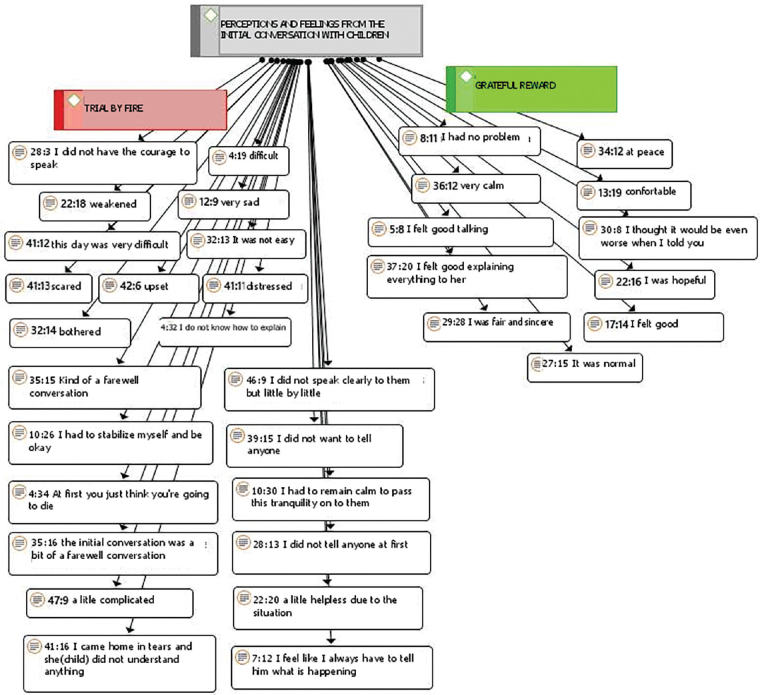
Parents’ perceptions and feelings in the initial conversation with their children between 3 and 12 years old about cancer diagnosis.

Figure 1 presents parents’ perceptions and feelings about the process of communicating the diagnosis to their children. The numbers contained in the rectangles, such as (28:3), express the document corresponding to participants inserted in the ATLAS.ti 8.0^R^ software and the selected content, respectively.

Parents’ perceptions and feelings about the process of communicating the diagnosis to their children were demarcated by positive statements, allocated to the right, and negative statements, allocated to the left, as shown in Figure 1, generating the “Grateful reward” and “Trial by fire” categories.

There were statements with a mixture of feelings of physical and emotional pain and, in others, relief when talking about the disease. *(...) it was difficult to talk to them... they have no idea what could happen, but they understand that I am sick (...). Today, I say that the tumor is gone. (...) my hair is already growing, I say everything is fine, but it’s still very difficult because they ask so often. They always remember, they always ask* (P1, 3- and 5-year-old daughters).

Family histories can lead to greater worry and increased stress when communicating with their child. *(...) he just stayed quiet, didn’t say anything, just looked at me. Afterwards, he became worried about me. He thought I was going to die because his aunt died, so he thought I was going to die (...). It was good that I spoke* (P26, 12-year-old son).

**Figure 2:**
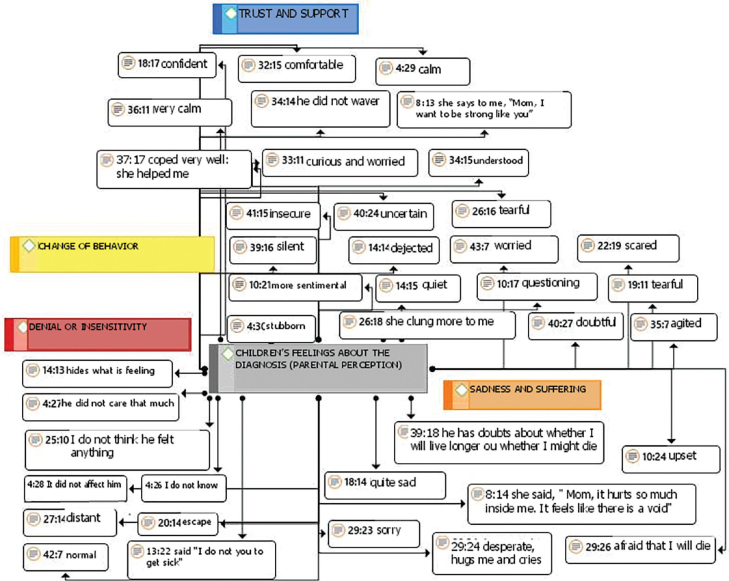
Children’s feelings from parents’ perspective based on communication about cancer diagnosis.

In parents’ perception, the communication of cancer diagnosis generated several feelings in their children that could be perceived at the time and, also, later. Figure 2 shows the categories in colored rectangles and the corresponding recording units.

The “Trust and support” category came, for the most part, from parents who had help from their spouse or other family members (grandparents, uncles or older brothers) when communicating their diagnoses to their children.

Participants indicated that children’s age, the cognitive, behavioral and attitudinal responses that the children expressed and parents’ own lack of familiarity with cancer influenced the dialogue. *(...) he doesn’t understand, I just say I have a pain in my stomach. Not that I’m a closed person, I’m a very open person, but I think there are things that are unnecessary to say because you won’t understand, and then I won’t know how to explain, so I prefer to go as basic as possible* (P27, 5-year-old son). *(...) she was sad, she talked to her teacher at school about me and the teacher also talked to her. She started to understand more and take it more in stride, she’s not so sad* (P15, 7-year-old daughter). *(...) he’s not very open. (…) he doesn’t touch on the subject, he doesn’t talk. I told him, he didn’t ask. Sometimes, when I come back from chemotherapy, he just asks if I’m okay, if I need anything.* (P39, 12-year-old son).

**Figure 3:**
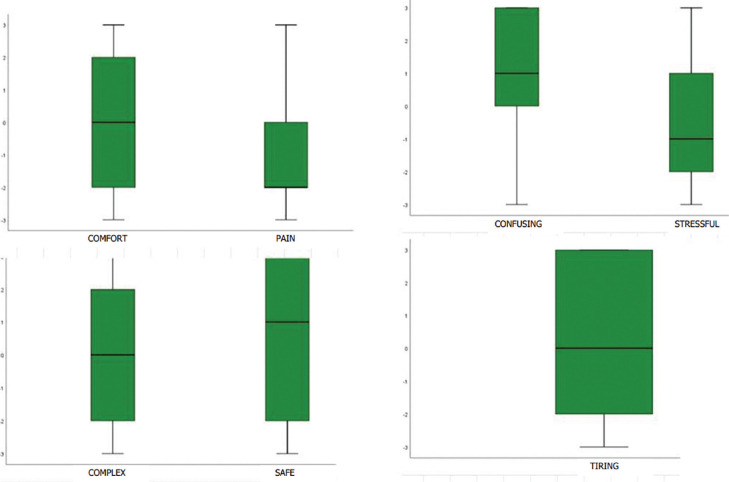
Graphics with adjectives that represent the predominance of parents’ feelings when talking to their children about cancer diagnosis.

In the search for validity of the data already obtained, parents selected the adjectives that represented the feeling experienced when communicating with their child about cancer diagnosis. EDS results were 73.1% painful, 58.5% neutral to simple, 51.2% safe, 53.7% clear, 53.6% stressful, and 51.3% neutral to not tiring, as illustrated in Figure 3.

The quantitative results reinforced the superiority of negative feelings, influencing the attitude of communication with their children about cancer diagnosis, as expressed in the “Trial by fire” and “Sadness and suffering” categories. On the other hand, they also expressed feelings that reproduced the findings of the “Grateful reward” and “Confidence in support” categories, characterizing them as safe, clear, tending to be simple and not tiring.

## DISCUSSION

The methodological design with data triangulation allowed us to reveal the perceptions and feelings of the process of communicating parental cancer to their children in a convergent and complementary way between quantitative and qualitative data in two different institutions, with similar sociodemographic profiles of participants.

The experience was characterized as negative in most experiences, generating an impact on children’s behaviors according to parents’ perception. The association of semi-structured questions with semantic differential questions offered a very complete and complex overview of how communicating a parental cancer diagnosis to children between 3 and 12 years old can be stressful and frightening for parents, even when there is social support.

In the final composition of the sample, female figured prominently, diagnosed with breast cancer and in young adults, results that corroborate the epidemiological data in which breast cancer predominates among women and tends to affect age groups that precede old age^(1,16)^.

The Global Cancer Observatory (GLOBOCAN), linked to the National Cancer Research Agency (IARC), published data on the growth of cancer in a young population aged 20 to 39 years. The document highlights the needs for prevention and intervention with effective actions, especially in developing countries and with a low-income population^(18)^.

Cancer in young adults proves to be aggressive and with higher rates of cell differentiation and biological factors that lead to an unfavorable prognosis, which highlights the need for attention when making decisions about actions for causes related not only to diagnosis, but also to screening and prevention^(2,18)^.

This scenario requires multidimensional care related to the control of malignant neoplastic disease and the psychosocial aspects involved with the illness, considering the social roles they play, life projects temporarily interrupted and, sometimes, constant concern for their own offspring, when there are siblings and children as possible heirs of genetic anomalies^(19,20)^.

Communication in the family constitutes a system in which each individual’s behavior is a factor and product of others’ response. The results depend less on initial conditions and more on the effective communication process in the family system that leads to social adaptation and favors resilience, according to social environment demands and mental health promotion^(20,21)^.

Communication strategies for children to cope with parental cancer involve open dialogue and exposure of feelings as well as participation in care with simple actions such as bringing a glass of water or bringing a blanket. Communication and participation help to alleviate anxiety and prepare children for any changes that may occur during treatment, such as hair loss, mood changes, episodes of nausea and vomiting, intense pain, crying and sadness^(19)^.

In the clinical profile of respondents, regarding treatments, the majority were undergoing antineoplastic chemotherapy and practically the same proportion had already undergone surgery, forming the two main interventions for treating many cancers^(22)^.

Among the participants, a minority were using a fully implanted central catheter, which represents safety and comfort for patients using intravenous antineoplastic chemotherapy and which, unfortunately, is not yet a reality in SUS oncology care. The use of clinical artifacts, such as catheters, stomas and skin expanders, was mentioned, but was restricted to a third of the sample. The artifacts conform to demands in health education for the multidisciplinary team, as they require adaptation and resilience. In this situation, the offspring may, at some point during treatment, be involved and feel like they are participating in care, understanding parental limitations^(23)^.

The majority of respondents had an ECOG between 0 and 1, with no or little limitation of activities of daily living, demonstrating satisfactory clinical conditions for implementing antineoplastic therapies with curative intentions and which tend to respond more quickly and effectively to the adverse effects of treatments^(16,24)^.

Of the participants who informed their children of parental cancer, the majority did so within the first week. Studies show that, at the beginning of diagnosis and treatment, parents tend to spare their children from bad news. However, over time, parents’ difficulties in coping with lack of dialogue with their children can have emotional effects on the offspring, such as depression and anger due to a lack of clarification of the reality they are experiencing^(21,25)^.

It is clear that parents often feel unable to face their own feelings and find themselves with the responsibility of also having to deal with their child’s pain. The inability to believe in the ability to cope can lead to concealment of information and emotions from everyone involved: parent with cancer, partner and offspring^(4,26)^.

Considering the number of perceptions and feelings in the “Trial by fire” category, it is worth highlighting the importance of understanding and supporting health teams regarding ambivalent feelings in the family experience of serious health problems, with educational approaches that can reflect on the relationships between patients and staff and patients and their families^(4)^.

In an Australian study, in which doctors caring for cancer patients with minor children participated, the challenges of conducting treatment by the medical team were explored. It was concluded that the ideal is to maintain a therapeutic stance so that patient-parents feel comfortable talking about their anxieties and concerns. Professionals reflected on the difficulty of dealing with patients’ situation, because ideally, in professional interactions, emotional support should be included, openness for parents to express concerns about their own abilities to care for children and the quality of communication that is established in the family^(21)^.

From the SR perspective, cancer is linked to negative adjectives and, therefore, positive responses, which express hope, can be surprising, as seen in the “Grateful reward” category. Parents, when talking about the disease with their children, identified feelings of trust and support^(12,13,16)^.

The “Sadness and suffering” category, perceived by parents in their children, shows the delicacy of the moment, which can also be induced by parents’ stance and attitude towards the gravity and complexity of the situation. Parents need experience in communication processes with health professionals in a clear, frank and respectful way so that the same process can be experienced in the relationship with their child^(7)^.

The “Trust and support” category, perceived by parents, is due to external help when communicating from family members, such as partners, grandparents, siblings and uncles. The family plays a fundamental role in society as a suitable institution with its particularities regarding beliefs, feelings, culture and values, contributing to the collectively constituted SR, which can help in communicating parental illness, since members feel are understood and supported^(24)^.

Data that corroborate the findings were obtained in a German study, in which one of the objectives was to assess the impact of parental cancer on children under 18 years of age. The results showed that the positive impact exists in smaller proportions than the negative ones, but they are reflected in children, as they demonstrate greater helpfulness, dedication to the family, self-confidence and responsibilities, in addition to better academic performance. More than half of participating parents reported needing support from grandparents in coping with the disease and caring for their children, help with household activities, financial and emotional support, among other demands^(3)^.

Communication with children in cases of parental cancer is still a challenge, but there are already initiatives for intervention and monitoring studies in different parts of the world, and the results have shown effectiveness in the behavior of parents with cancer and their children, especially when family relationships are permeated with insecurity, sadness and anguish, configuring the need to monitor emotions and introduce psycho-oncological interventions for all family members^(27)^.

The “Change of behavior” category exemplified that parents should be aware of the different manifestations of behavior in the continuum of cancer illness. These children/adolescents may witness, for years, various stages of their parents’ illness and, during this journey, they may miss a targeted approach, with clear language so that they can understand and overcome the multiple difficulties^(28)^.

The “Denial or insensitivity” category deserves special care, as it runs the risk of devaluing situations that are as complex as the quality of interpersonal interaction between parents and children. Children have a peculiar way, depending on the phase of their psychomotor development and their age, to understand complex issues such as cancer, while adolescents require direct and open communication^(20)^.

Denial can be understood as a protective reaction to suffering on the part of children and adolescents, or as an unexpected response that is difficult to interpret, in isolation, considering the perception of insensitivity/indifference. To address the issue, it is necessary to equip parents with educational strategies that take into account the particularities of the illness situation^(28,29)^.

Cancer is still a taboo and brings with it the population’s prejudice and lack of information about early diagnosis and the need to seek medical care. In general, people are afraid of knowing the result of diagnosis and know little about treatment in a practical and up-to-date way. However, there are constant advances in oncology, and cancer diagnosis requires shared discussions about treatments and prognoses that, despite being promising in many cases, bring difficulties in the coexistence of this family, oscillating between overestimation or underestimation of care, at all stages of the process, including survival^(29,30)^.

Given the research results, there was a lack of national studies on the topic, limiting possible comparisons with other studies on parental communication regarding parental cancer with children aged between 3 and 12 years. From a sampling perspective, the majority of respondents were female, and in future studies, a balanced sample for both sexes or inclusion of genders would also be interesting.

The validation of the discursive responses was not carried out, as explained, as well as that of the EDS descriptors used, despite its construction having followed the guidelines of the creators. Another factor considered limiting was the time elapsed from diagnosis to participation in the interview, which was up to 24 months, which may have made it difficult to remember in detail the events at the time of communicating about the diagnosis with their children.

The implications for practice arising from this investigation bring the need for investments in strategies that include family care, globally, in multidisciplinary care, especially in nursing consultations due to capacity for multidimensional assessment.

## CONCLUSION

The majority of participants were young adults, with breast cancer, living with a partner, undergoing multimodal treatment, in full functional clinical conditions or with very little impairment and with offspring restricted to one child. The disclosure of the parental diagnosis to children occurred at different times, most of them in the week in which cancer diagnosis was defined, regardless of the age group of children.

The moment when diagnosis was revealed to children was mostly described with negative perceptions and feelings, using words such as goodbye, anguish, discomfort with conversation, fragility, sadness, fear of death. However, it was observed that some parents had positive perceptions and feelings when using the words trust, tranquility, feeling of well-being and hope.
